# The ASME-speller: 30-class auditory brain-computer interface speller using stream segregation and the QWERTY layout

**DOI:** 10.3389/fnhum.2026.1807535

**Published:** 2026-05-21

**Authors:** Simon Kojima, Shin'ichiro Kanoh

**Affiliations:** 1 Graduate School of Engineering and Science, Shibaura Institute of Technology, Tokyo, Japan; 2 College of Engineering, Shibaura Institute of Technology, Tokyo, Japan

**Keywords:** auditory BCI, BCI speller, brain-computer interface, deep learning, electroencephalography, event-related potential, machine learning, stream segregation

## Abstract

**Introduction:**

This study presents the ASME-speller, a novel 30-class auditory brain-computer interface (BCI) speller system that combines auditory stream segregation with the familiar QWERTY keyboard layout to facilitate intuitive and visionfree communication.

**Methods:**

In the ASME-speller, three distinct auditory streams are presented simultaneously, each corresponding to a row on the QWERTY keyboard. The low-, middle-, and high-frequency streams represent the bottom, middle, and top rows, respectively. Within each stream, alphabet letters and selected symbols are repeatedly presented as spoken voice stimuli. Users are instructed to focus exclusively on the stream corresponding to the row containing the target letter and to selectively attend to that letter within the stream. By leveraging the QWERTY layout and auditory stream segregation, the proposed approach enables users to restrict their attentional focus to a subset of letters by directing selective attention to auditory streams, while the mapping between QWERTY rows and stream pitch facilitates intuitive letter selection. We conducted online experiments with ten healthy participants to evaluate system performance.

**Results:**

The ASME-speller achieved an average classification accuracy of 0.76 and an average information transfer rate (ITR) of 2.16 bits/min. Excluding one participant whose EEG data contained excessive artifacts, these values improved to 0.84 and 2.40 bits/min, respectively. Post-hoc analyses further examined the effects of preprocessing parameters, classification pipelines, and early stopping strategies. Among four pipelines tested, a linear discriminant analysis (LDA) combined with dynamic stopping demonstrated the most robust performance across participants (accuracy of 0.80 and ITR of 4.76 bits/min). For the best participant, a deep learning model (EEGNet4,2) with dynamic stopping achieved accuracy of 1.0 with ITR of 14.44 bits/min.

**Discussion:**

Compared to previous auditory BCI spellers, the ASME-speller demonstrates performance comparable to existing systems, while offering advantages in terms of simplicity, requiring only standard headphones and no visual support. These findings demonstrate the feasibility of the ASME-speller and pave the way toward practical auditory BCI applications for communication.

## 1 Introduction

The brain-computer interface (BCI) enables direct communication between the brain and a computer (Wolpaw et al., 2000, 2002). Since it does not require any limb movement, patients with amyotrophic lateral sclerosis (ALS), locked-in syndrome (LIS), or other severe neurological disorders can use such systems to control external devices (Cipresso et al., 2012; King et al., 2013; Marchetti and Priftis, 2014; Wang et al., 2025; Chaudhary et al., 2021; Zhang et al., 2017). Electroencephalography (EEG) is widely employed for BCI due to its high temporal resolution and portability.

Various applications of BCIs have been explored; however, surveys of patients with locked-in syndrome have revealed that over 90% prioritize direct communication as the most desired function of a BCI system (Branco et al., 2023). Consequently, many BCI speller applications have been proposed (Kundu and Ari, 2022). Among them, numerous visual stimulus-based BCI spellers have been developed. One of the most well-known examples is the P300 speller, first introduced by Farwell and Donchin (1988). Additionally, BCI speller systems utilizing steady-state visual evoked potentials (SSVEP) (Li et al., 2021) and code-modulated visual evoked potentials (c-VEP) (Martínez-Cagigal et al., 2021) have achieved high accuracy and information transfer rate (ITR). Nevertheless, it is known that patients with severe neurological disorders often experience difficulties with gaze control, making vision-based systems less suitable for this population (Choi et al., 2023). Moreover, in the broader context of human-computer interaction (HCI) systems, including those intended for healthy users, it is undesirable for vision, which is one of the most essential sensory modalities in daily life, to be fully occupied by a BCI task.

In contrast, auditory BCIs can be used by individuals with visual impairments and do not interfere with vision. To address the limitations of visually based systems, several auditory BCI spellers have been proposed. One of the earliest approaches was introduced by Furdea et al. (2009), which is inspired by the visual P300 matrix speller (Farwell and Donchin, 1988), in which 25 alphabet letters were arranged in a 5 × 5 matrix. Each letter was represented by two auditory stimuli corresponding to its row and column, presented as spoken number words. Letter selection was performed in two steps: identifying the target row in the first step and the target column in the second. Klobassa et al. (2009) proposed a similar approach using a 6 × 6 matrix that included all 26 letters and additional symbols, replacing spoken numbers with environmental sounds. Schreuder et al. (2011b) applied the AMUSE paradigm (Schreuder et al., 2010) to a speller application, where letters were divided into six groups and presented via loudspeakers spatially arranged around the user.Target selection was also achieved through a two-step process: first selecting the target group, then selecting the letter within that group. A similar system has also been tested using Japanese syllables. This system covered 25 of the 50 Japanese syllables (Chang et al., 2014). Höhne et al. (2011) proposed the PASS-2D system, a nine-class auditory speller inspired by the T9 input method used in mobile phones, combining pitch and spatial cues for stimulus coding. The system allowed users to select a letter within a single step. In addition, it incorporated predictive text functionality: when the desired word appeared in the prediction list, users could confirm the word directly; otherwise, they continued by spelling additional letters.

Subsequently, Höhne and Tangermann (2014) introduced CharStreamer, which divided the alphabet into three groups and presented each group from a distinct spatial direction, allowing target letter detection within a single step. Kleih et al. (2015) presented the WIN-speller, which first played sequences of words. The user selected a word containing the target letter, after which the letter was identified from within that word, requiring two steps per letter. More recently, Markovinović et al. (2022) proposed a system in which spoken letters were presented in sequence, and the target letter was inferred based on classifier outputs. If the estimated probability of a letter exceeded a predefined threshold, the letter was selected after a single step; otherwise, a second step was conducted using the five most likely candidates. However, this system was limited to inputting only 22 out of the 26 alphabet letters.

To the best of our knowledge, auditory-only BCI spellers proposed so far are limited to the systems described above and their variants. With the exception of Markovinović et al. (2022), most of these systems were proposed before 2015, and few new paradigms based solely on auditory stimuli have been introduced in recent years.

In contrast, many hybrid BCI spellers incorporating auditory stimuli have been proposed recently. For example, audiovisual spellers have been introduced in Oralhan (2019), Lu et al. (2019), and Belitski et al. (2011). In the study by Lu et al. (2019), the system using audiovisual stimuli achieved higher accuracy than a system using visual stimuli alone. Moreover, Lu et al. (2019) and Belitski et al. (2011) reported that combining auditory and visual stimuli improved performance compared with using either auditory-only or visual-only stimulation. In addition, systems combining auditory and tactile stimuli have been proposed in Zhang et al. (2020) and Jiang et al. (2019). These studies reported that combining both stimuli improved performance by more than 30% compared with using either auditory-only or tactile-only stimulation. Thus, hybrid BCI spellers using auditory stimuli have been reported to achieve higher accuracy than systems relying solely on auditory stimulation. However, systems based exclusively on auditory stimuli remain advantageous in that they can be used by users with visual impairments and require only widely available devices such as headphones or speakers, unlike tactile stimulation systems that require specialized hardware. Therefore, auditory-only BCI spellers remain important for enabling users with diverse backgrounds to select systems that best suit their individual needs.

While some auditory BCI spellers have been reported, they generally suffer from one or more of the following limitations: (i) more than two steps are required to select a single letter; (ii) the mapping between auditory stimuli and letters is not intuitive, requiring visual support or imposing a high memory load; and (iii) multi-channel audio setups are necessary, which are cumbersome and may exclude users with unilateral hearing loss.

By leveraging auditory stream segregation, these limitations can potentially be overcome, enabling the development of the ASME-speller—a novel auditory BCI speller that utilizes stream segregation.

### 1.1 ASME paradigm and ASME-speller

ASME (Auditory Stream Segregation Multiclass ERP) is an auditory BCI paradigm based on auditory stream segregation, first proposed by Kanoh et al. (2010). Auditory stream segregation is a perceptual phenomenon whereby alternating auditory stimuli, such as an ABABAB… sequence, are perceived as two separate streams (e.g., AAA… and BBB…). In the ASME-BCI, oddball sequences are embedded within such streams. Users direct their selective attention to one of the streams, as well as to specific stimuli within that stream. Event-related potentials (ERPs), including the P300, elicited by the attended stimuli, can then be detected and used to infer the user's focus of attention.

With ASME-based BCI, multiclass classification can be achieved using only monaural audio; that is, it requires only a single audio channel. The authors proposed ASME-based BCI systems with two (Kanoh et al., 2008, 2010), three (Kojima and Kanoh, 2024a), and four classes (Kojima and Kanoh, 2024b). In particular, the four-class system demonstrated that multiple target stimuli could be embedded within a single stream, enabling multiclass detection with a limited number of streams.

**Figure 1:**
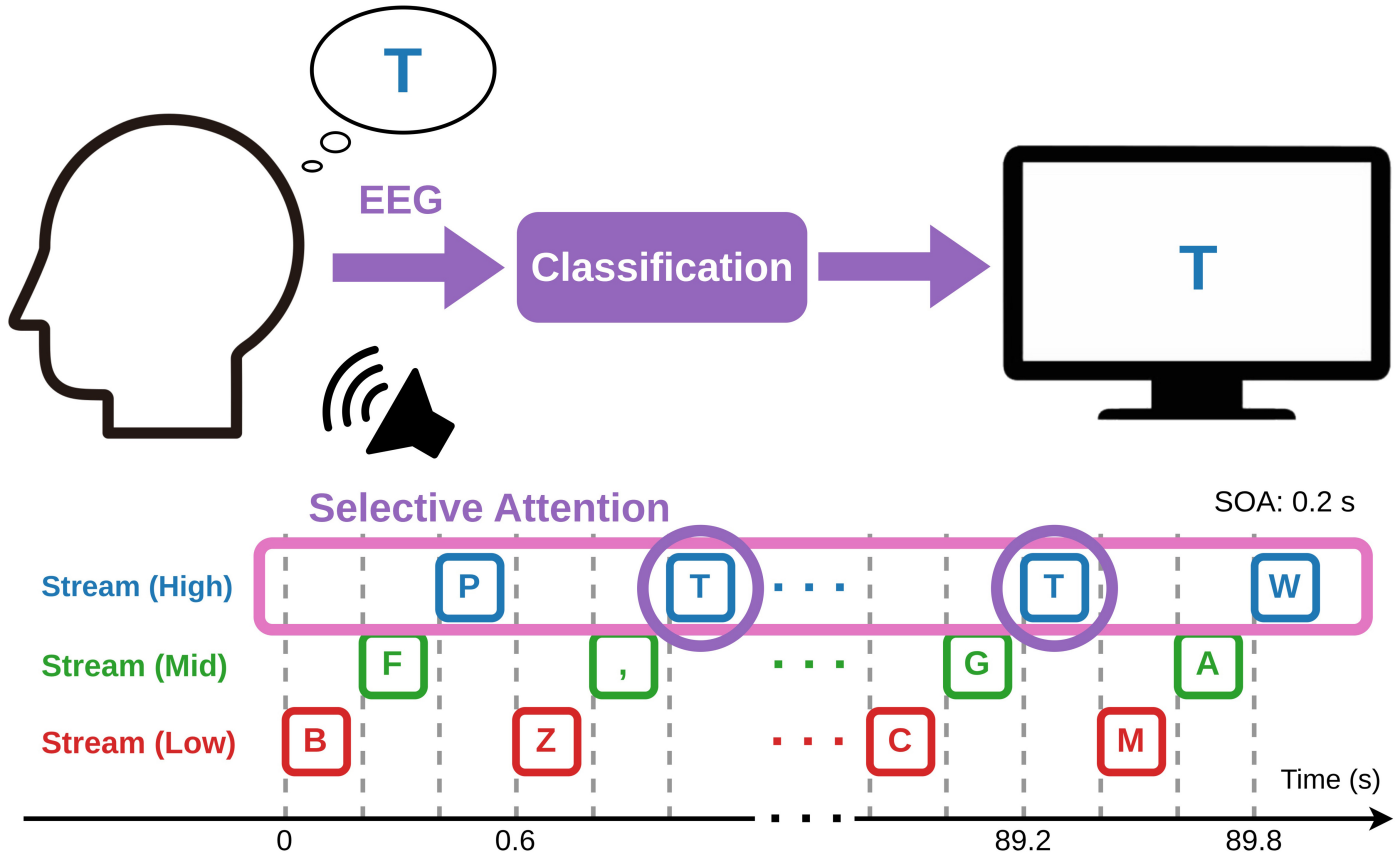
Conceptual diagram of the ASME-speller BCI. Voice stimuli corresponding to alphabet letters are presented repeatedly in three auditory streams, each assigned to one QWERTY row: **top** (high frequency), **middle** (mid frequency), and **bottom** (low frequency). For example, to type the letter “T,” which belongs to the top row of QWERTY layout, the user attends to the high-frequency stream and pays selective attention to the vocal stimulus “T.” Event-related potentials (ERPs) are elicited in response to the attended stimulus, and the BCI system detects the ERP to estimate the intended target letter. When the number of sequences is 15, the trial length is approximately 90 s.

**Figure 2:**
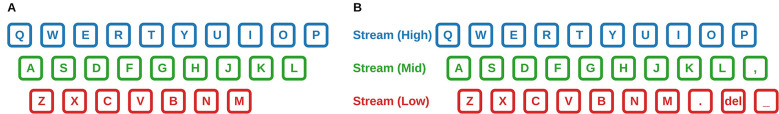
**(A)** The standard QWERTY layout. **(B)** The letters used in the experiment and their corresponding auditory stream.

To realize a practical speller BCI application based on the ASME paradigm, we propose an auditory BCI speller system that maps letters onto streams corresponding to the QWERTY keyboard layout, referred to as the ASME-speller (see Figure 1 for the conceptual diagram). The QWERTY layout (Figure 2A) is one of the most widely used keyboard layouts for computers and smartphones, and many users are expected to already be familiar with it (Yasuoka and Yasuoka, 2011). Since all 26 alphabet letters can be mapped onto three rows of keys, the entire QWERTY layout can be naturally represented using a three-stream ASME paradigm. In the ASME-speller, the top, middle, and bottom rows of the keyboard are assigned to auditory streams with high, middle, and low frequency bands, respectively. Within each stream, each letter is presented as a spoken voice stimulus.

Figure 2 illustrates the mapping between the QWERTY layout and the corresponding tone streams. In this speller task, the user first identifies the row in which the intended letter is located on the QWERTY layout and attends to the corresponding stream. Within that stream, the user selectively focuses on the target letter stimulus. For example, when the user intends to input the letter “T,” they recognize that “T” belongs to the top row, attend to the high-frequency stream, and focus on the “T” sound stimulus. Consequently, ERP components such as the P300 are elicited by the attended stimuli (Kanoh et al., 2008, 2010; Kojima and Kanoh, 2024a), and the target letter can be identified by detecting these ERP responses using machine learning methods. In the ASME-speller: (i) the target letter can be selected in a single step; (ii) the mapping between auditory stimuli and letters is intuitive and does not require visual support if the user is already familiar with the QWERTY layout; and (iii) all stimuli can be presented through a monaural audio channel. Thus, it offers a potential solution to limitations observed in previously proposed auditory BCI spellers.

Building on this design, spoken alphabet stimuli are presented within multiple perceptually segregated auditory streams, each corresponding to a row of the QWERTY layout. This structure allows the user to first restrict their attentional focus to a subset of letters by attending to a specific stream, and then select the desired letter by focusing on the corresponding stimulus within that stream. Furthermore, because the vertical arrangement of QWERTY rows is systematically mapped to the pitch of the auditory streams, users can intuitively direct attention to the appropriate stream.

As a pilot study, we tested the ASME-speller using 15 alphabet letters (Kojima and Kanoh, 2024c). It achieved a letter selection accuracy of 0.73 and an information transfer rate (ITR) of 3.78 bits/min, demonstrating the feasibility of the ASME-speller. However, the number of available classes was still limited and insufficient to cover all 26 alphabet letters. Additionally, the experiment was conducted in an offline manner; that is, classification was performed after the EEG recording session. Thus, it remained unclear whether online spelling could be realized with the ASME-speller.

In this study, we extend our previous work and introduce the ASME-speller, a 30-class auditory BCI speller system that enables full alphabet letters (and some symbols) input based on auditory stream segregation. We conducted online experiments with healthy participants to evaluate its feasibility as a practical speller system. In addition, we performed *post-hoc* analyses to investigate the extent to which performance can be improved by optimizing preprocessing parameters, classification pipelines, and employing early stopping strategies.

In summary, we propose the ASME-speller, a novel 30-class auditory BCI speller that leverages auditory stream segregation and the familiar QWERTY keyboard layout to enable intuitive and effective communication. We demonstrate its feasibility through online experiments with healthy participants, showing that the system achieves classification accuracy and information transfer rate (ITR) comparable to those of previously reported auditory speller systems.

Furthermore, multiple classification pipelines and early stopping strategies were systematically evaluated, and configurations showing robust performance across participants were identified based on the current dataset.

## 2 Methods

### 2.1 Participants

Ten native Japanese-speaking participants (aged 21–28 years; mean age = 23.4; four female) participated in this study. The study protocol was approved by the Review Board on Bioengineering Research Ethics of the Shibaura Institute of Technology and conducted in accordance with the Declaration of Helsinki. Prior to the experiment, all participants were provided with information both orally and in writing. Written informed consent was obtained from all participants. None of the participants had any known cranial nerve disorders or hearing impairments.

### 2.2 EEG measurement

EEG was recorded from 64 channels (Fp1, Fp2, AF7, AF3, AFz, AF4, AF8, F7, F5, F3, F1, Fz, F2, F4, F6, F8, FT9, FT7, FC5, FC3, FC1, FCz, FC2, FC4, FC6, FT8, FT10, T7, C5, C3, C1, Cz, C2, C4, C6, T8, TP9, TP7, CP5, CP3, CP1, CPz, CP2, CP4, CP6, TP8, TP10, P7, P5, P3, P1, Pz, P2, P4, P6, P8, PO7, PO3, POz, PO4, PO8, O1, Oz, and O2), along with two electrooculogram (EOG) channels (vertical and horizontal), using the BrainAmp system (Brain Products, Germany). Signals were sampled at 1, 000 Hz using passive Ag/AgCl electrodes (EasyCap, Germany). Electrodes were placed according to the extended 10–20 system. The reference and ground electrodes were placed on the right and left mastoids, respectively. All signals were recorded and streamed using the Lab Streaming Layer (LSL) (Kothe et al., 2025).

### 2.3 Stimuli

The 26 alphabet letters (A to Z) and four additional symbols (comma, delete, space, and period) were used in this experiment. Each voice stimulus was generated using Amazon Polly, a cloud-based text-to-speech service provided by Amazon Web Services (AWS). The voice stimuli corresponding to the top, middle, and bottom rows of the QWERTY layout were synthesized using the voice IDs *Ruth* (female), *Kevin* (male child), and *Joey* (male), respectively. Different voice IDs were assigned to the top, middle, and bottom rows so that the resulting streams could be perceived as having high, middle, and low pitch, respectively. See Supplementary Table S1 for the prompts used to generate each stimulus. The pitch of the voice stimuli corresponding to the top and bottom rows was further adjusted by +3 and −3 halftones, respectively. The symbol *comma* was assigned to the stream with a middle frequency, while *space, delete*, and *period* were assigned to the stream with a low frequency. Figure 2B shows the mapping between each letter and its corresponding stream. All audio files used in the experiment are provided in the dataset (see data availability statement).

### 2.4 Experimental design

Each participant completed one EEG measurement session consisting of nine runs. The first six runs were offline runs, in which data were collected for training the classification model. The last three runs (runs seven to nine) were online runs, where the recorded signals were analyzed and classified in real time, and the participants received BCI output feedback immediately after each trial.

In this study, a trial does not refer to a single stimulus presentation or the classification of a response to a single stimulus. Instead, a trial denotes the entire process in which the user maintains selective attention to one target letter while multiple stimulus sequences are presented, and the system classifies the responses to determine the selected letter. Each run consisted of five trials. Supplementary Table S2 shows the target letters used in each trial and run. In the offline runs, 30 trials were conducted, in which each of the 30 letters was presented once as the target. In the online runs, 15 trials were conducted, in which 15 letters were tested as targets. However, the system output was always selected from the full set of 30 letters, i.e., the system performed a 30-class decision in every trial.

The bottom half of Figure 1 shows an example timeline of a trial. Each trial consisted of 15 sequences. In each sequence, the letter stimuli were presented once per stream, following the order of the QWERTY keyboard rows: bottom, middle, and top. These corresponded to the low-, middle-, and high-frequency auditory streams, respectively. Within each stream, the order of letters was pseudorandomized. As a result, each trial contained 450 stimuli and lasted approximately 90 s. The stimulus onset asynchrony (SOA) was set to 0.2 s. All sound stimuli were delivered via Fireface 802 (RME, Germany) and presented through headphones (MDR-EX800ST, Sony, Japan).

During the experiment, participants sat in a comfortable chair in a soundproof, electromagnetically shielded room. A display was placed in front of each participant. At the beginning of each trial, the target letter was indicated by displaying it on the screen, followed by the stimulus presentation. All participants were familiar with the QWERTY layout; therefore, no visual support for mapping between letters and auditory streams was provided. During each trial, participants selectively attended to the auditory stream corresponding to the target letter and focused attention on the target stimulus. In the online runs, both the target letter and the letter predicted by the BCI system were presented as feedback at the end of each trial.

Resting-state EEG was recorded before and after the ASME-speller task. During these recordings, EEG data were collected for one minute each while the participants had their eyes open and closed. Additionally, prior to the ASME-speller task, two runs of an auditory oddball task were conducted. In each oddball run, 500 Hz (non-target) and 1, 000 Hz (target) tones were presented pseudorandomly 250 and 50 times, respectively, and participants were instructed to pay attention to the target stimuli.

### 2.5 Analysis performed during the online run

The following analysis was performed during the online runs. First, a classification model was trained using the EEG data recorded in the offline runs. Artifact removal was not applied to the data used for model training or during online runs, in order to keep the processing pipeline simple and to focus on evaluating the feasibility of the ASME-speller in a minimal configuration. Only 64 EEG channels were used for classification. A second-order Butterworth filter (1–40 Hz) was applied in the forward direction to all EEG data from the offline runs. Epochs were extracted from −0.1 to 1.0 s relative to stimulus onset. The mean amplitudes were calculated in ten non-overlapping 0.1-second intervals from 0 to 1.0 seconds and used as classification features. The resulting feature vector had a dimension of 640 (64 channels × 10 intervals).

For classification, linear discriminant analysis (LDA) with Ledoit-Wolf covariance shrinkage (Blankertz et al., 2011; Ledoit and Wolf, 2004) was used. The classification task of LDA was a binary classification to distinguish responses to target vs. non-target stimuli. The classifier was trained so that responses to target stimuli yielded positive classifier outputs. In total, 13, 500 samples (450 targets and 13, 050 non-targets) were used for training.

Once the classifier was trained, the online runs were initiated. For EEG data streamed via LSL during the online runs, a preprocessing pipeline identical to that used in offline runs (i.e., bandpass filtering and the feature extraction) was applied in real time. The response to each letter stimulus was then classified, and the classifier output (i.e., the distance from the classification hyperplane) was recorded. After classifying all 450 stimuli in a trial, the mean classifier output was computed for each class (i.e., letter or symbol). The class with the highest mean output was selected as the final output of the BCI system. This final output was displayed on a screen in front of the participant as feedback.

The dataset exhibited a class imbalance of 1:29. However, the LDA classifier used in this study was based on the binary Fisher discriminant direction $w=S_{w}^{-1}\left(m_{1}-m_{0}\right)$, where *S*_*w*_ denotes the pooled within-class covariance matrix, and *m*_1_ and *m*_0_ denote the mean feature vectors of the target and non-target classes in the training data (Bishop, 2006). This projection is determined by the difference between the class means and the pooled within-class covariance, and does not explicitly depend on the class prior probabilities. Along this direction, the projected value of the target-class mean is larger than that of the non-target mean ($w^{⊤}m_{1}>w^{⊤}m_{0}$). In addition, in the classification procedure used in this study, the classifier output was defined as $d_{i}=w^{⊤}x_{i}+b$ (where *b* denotes the bias term). For each trial, the mean value of *d*_*i*_ was computed for each candidate class, and the class with the largest mean was selected as the final output. Therefore, the classification was not performed using a fixed threshold based on the bias term. Instead, it relied only on the relative magnitudes of *d*_*i*_ within each trial. As a result, the class prior probabilities do not directly determine the final trial-wise decision in this framework, although the class imbalance may still influence parameter estimation through the covariance matrix and class means.

Performance was evaluated using classification accuracy and information transfer rate (ITR). Classification accuracy was assessed at the trial level, defined as whether the target letter was correctly selected at the end of each trial. The accuracy was computed as the proportion of correctly selected target letters across all trials. ITR quantifies the amount of information communicated by the system per unit time (Rao, 2019). The ITR was computed using the following equations (Schreuder et al., 2010), where *N* is the number of classes, *P* is the classification accuracy, *V* is the classification speed in trials/min, *R* is the ITR in bits/trial, and *B* is the ITR in bits/min:


$$\begin{array}{l} R=\log_{2}\left(N\right)+P\log_{2}\left(P\right)+\left(1-P\right)\log_{2}\left(\frac{1-P}{N-1}\right) \end{array}$$ (1)

$$\begin{array}{l} B=V\cdot R \end{array}$$ (2)

The trial duration used for ITR calculation (i.e., the time required to select one letter) was defined as *T* = (*N*_*s*_−1) × *SOA*+*T*_*max*_, where *N*_*s*_ denotes the number of presented stimuli and *T*_*max*_ denotes the EEG epoch length (in seconds). Thus, *T* is expressed in seconds. In the online experiment, a total of 450 stimuli were presented with an SOA of 0.2 s, and the EEG epoch length used for classification was 1.0 s. Therefore, the trial duration was 90.8 s.

We estimated 95% confidence intervals for the classification accuracy and ITR for each participant. The confidence intervals for classification accuracy were computed by treating accuracy as a binomial proportion (i.e., the number of correctly classified trials out of the total number of trials) and using the Wilson score interval. The confidence intervals for ITR were obtained by converting the lower and upper bounds of the accuracy confidence intervals into the corresponding ITR values. Furthermore, the 95% confidence intervals for the mean classification accuracy and ITR across participants were estimated using bootstrap resampling at the participant level (10,000 iterations).

### 2.6 *Post-hoc* analysis

This section and the following sections describe the *post-hoc* analyses performed on the recorded EEG data. When filtering was applied, a second-order Butterworth filter was used in the forward direction, with the frequency range varying depending on the specific analysis. After epoch extraction, the data were downsampled to 128 Hz. EEG epochs were extracted from −0.1 to 2.1 s relative to the onset of each auditory stimulus. However, in some runs, the recording terminated before 2.1 s had elapsed after stimulus onset. To ensure consistent epoch lengths across trials, 5 s of zero-padding were added to the end of each run. For the ERP analysis (Section 2.7), data from subject 10 were excluded due to excessive noise, although this participant was included in the other classification analyses.

For the *post-hoc* analysis, Python 3.11, MNE-python (Gramfort et al., 2013), and scikit-learn (Pedregosa et al., 2011) were used.

### 2.7 ERP analysis

To visually inspect ERP responses, the following analysis was conducted separately for the data recorded in the offline and online runs.

First, EOG artifact removal was performed. A second-order Butterworth high-pass filter with a cutoff frequency of 1 Hz was applied in the forward direction. Principal component analysis (PCA) was applied to extract 15 components, followed by independent component analysis (ICA) (Hyvärinen et al., 2001; Winkler et al., 2015). The Pearson correlation coefficient was computed between each ICA component and each EOG channel, and for each EOG channel, the component with the highest Pearson correlation was identified as EOG-related.

For EEG data visualization, the ICA component marked as EOG was zeroed out. Then, a band-pass filter (1–40 Hz) was applied, and EEG epochs were extracted. ERP responses to target and non-target stimuli were averaged across epochs. Additionally, signed-*r*^2^ values Blankertz et al. (2011) were computed to assess the separability between target and non-target responses.

### 2.8 Effect of artifact removal

In the online experiment of this study, artifact removal (e.g., EOG-related artifacts) was not applied. To evaluate the impact of this design choice, we conducted an offline simulation in which ICA-based artifact removal was incorporated.

The ICA decomposition matrix and the components to be removed were estimated using the training data (runs 1–6), following the same procedure described in Section 2.7. The estimated ICA decomposition matrix was then applied to the online data (runs 7–9) to project the signals into the independent component space. After removing the identified components, the signals were reconstructed using the corresponding mixing matrix. The reconstructed signals were subsequently processed using the same classification pipeline as in the online experiment.

For performance evaluation, trial-wise letter selection accuracy was used. Statistical comparisons between the actual online results and the ICA-based simulation were conducted using the Wilcoxon signed-rank test.

### 2.9 Effect of class imbalance

In the design of the 30-class ASME-speller, a class imbalance of 1:29 was present. Specifically, the number of samples used for training the LDA classifier was *N*_*T*_:*N*_*nT*_ = 450:13050, where *N*_*T*_ and *N*_*nT*_ denote the number of samples in the target and non-target classes, respectively.

To evaluate the effect of this class imbalance in the training data on classification performance, we performed an analysis in which the class ratio of the training data was manipulated by sub-sampling the non-target class data from the offline runs (runs 1–6). First, for each participant, the EEG data were preprocessed as described in Section 2.6, and epochs in the time interval of 0.0–1.0 s relative to stimulus onset were extracted. The extracted interval was then divided into ten non-overlapping 0.1 s segments, and the mean amplitude within each segment was used as a feature. Next, for each participant, all target-class samples were retained, while non-target samples were randomly sub-sampled such that the ratio of non-target samples relative to *N*_*T*_ became 1, 5, 10, 20, and 29. Using the sub-sampled datasets, LDA classifiers were trained and applied to classify the online experimental data (runs 7–9). All samples in the online data were used without sub-sampling. Based on the classification results, the target letter in each trial was estimated, and the letter selection accuracy was computed. The sub-sampling procedure was repeated 10 times for each ratio, and the resulting letter selection accuracies were averaged to obtain the final performance for each ratio.

### 2.10 BCI simulation

In the online runs conducted during the experiment, the classification pipeline was selected as described above; however, it was not guaranteed to be optimal. Therefore, a *post-hoc* BCI simulation analysis was conducted to investigate how much performance could be improved by using alternative preprocessing parameters and machine learning pipelines.

In the online experiment, each trial lasted approximately 90 s, during which one letter was selected based on the accumulated EEG data. However, for practical BCI applications with higher ITR, reducing the trial duration is desirable. To investigate the impact of shorter trial durations on performance and to achieve a reduction in trial duration without substantially degrading accuracy, two early stopping strategies (static stopping and dynamic stopping) were applied and evaluated.

For the further analysis, only EEG channels were used for the BCI simulation.

### 2.11 Exploration of preprocessing parameters and classification pipelines

Before conducting the BCI simulation, the optimal preprocessing parameters were explored using only the data from the offline runs. Specifically, the following parameters were varied: the high-pass cutoff frequency, the low-pass cutoff frequency of the band-pass filter, and the epoch length (i.e., the time interval from stimulus onset). See Table 1 for the list of explored parameters. The parameters selected in this analysis were used in subsequent simulations. In addition to preprocessing hyperparameters, the following four classification models were evaluated: (1) LDA, (2) xdw+LDA, (3) xdwCov+TS+LDA, and (4) EEGNet4,2.

**(1) LDA:** From the extracted epoch data, mean amplitudes were computed in non-overlapping 0.1-second intervals from 0 s to the epoch length relative to the stimulus onset. These values were used as classification features. The resulting feature vectors were classified using shrinkage regularized linear discriminant analysis (Blankertz et al., 2011).
**(2) xdw+LDA:** First, the xDAWN spatial filter (Rivet et al., 2009, 2011) was applied to extract two xDAWN components. xDAWN is a spatial filtering method designed to enhance ERP signals by maximizing the signal-to-signal-plus-noise ratio. After extracting xDAWN components, the pipeline was identical to that in (1) LDA.
**(3) xdwCov+TS+LDA:** The xDAWN filter was applied to extract two components, and covariance matrices were then computed Barachant and Congedo, (2014). These matrices were classified using a Riemannian geometry-based pipeline. Covariance matrices were manipulated using Riemannian geometry, and the geometric mean of all matrices was computed on the Riemannian manifold. Each matrix was then projected onto the tangent space (TS) at the geometric mean, which is an Euclidean space (Yger et al., 2017). Shrinkage LDA was used for classification in the tangent space. For further details, refer to (Barachant and Congedo 2014). To implement this pipeline, a Python library pyRiemann (Barachant et al., 2025) was used.
**(4) EEGNet4,2:** EEGNet is a deep learning architecture specifically designed for EEG classification and is widely used in BCI research (Lawhern et al., 2018; Patel et al., 2023; Xu and Xia, 2023; Chevallier et al., 2024). The number of filters in the convolutional layers was set as follows: *F*1 = 4, *D* = 2, and *F*2 = 8. The dropout rate was set to 0.5. See Lawhern et al. (2018) for further details about the architecture. The model was trained using the AdamW optimizer (learning rate = 1 × 10^−3^, weight decay = 1 × 10^−2^) with a batch size of 64 for up to 500 epochs. A cosine annealing learning rate scheduler was applied (T_max_ = 500, ${\text{η}}_{\text{min}}=1\times 10^{-6}$). The loss function was weighted cross-entropy, where class weights were set to [1.0, 29.0], assigning a weight of 1.0 to non-target responses and 29.0 to target responses, to compensate for class imbalance. A Python library Braindecode (Schirrmeister et al., 2017) was used to implement this pipeline. Early stopping with a patience of 75 epochs was applied based on the validation loss to prevent overfitting. As a preprocessing step, each EEG channel data was normalized to have zero mean and unit variance. The normalization parameters were estimated from the training set and applied to the validation and test sets. The model with the lowest validation loss was saved and used for the final evaluation on the test set.

**Table 1:** Preprocessing parameters explored in Section 3.5.

Parameter	Explored values
Highpass cutoff (Hz)	0.1, 0.5, 1.0
Lowpass cutoff (Hz)	8, 15, 20, 40
Epochs Tmax (s)	0.5, 1.0, 1.5, 2.0

The high-pass and low-pass cutoff frequencies define the band-pass filter applied during preprocessing. Epoch length (Tmax) denotes the duration of EEG segments extracted relative to stimulus onset.

All classification models were evaluated using 3-fold cross-validation, which respected the boundaries of the six runs (see Supplementary Tables S3, S4 for fold definitions). Classification performance was evaluated using the area under the receiver operating characteristic curve (AUC). The AUC was computed at the single-stimulus level in this parameter exploration, i.e., using classifier outputs for each stimulus presentation.

### 2.12 BCI simulation with static stopping

Based on the results from Section 2.11, the following preprocessing parameters were used: highpasscutoff = 0.5 Hz, lowpasscutoff = 8 Hz, and epochs length = 2.0 s for LDA, xdw+LDA, xdwCov+TS+LDA pipelines. highpasscutoff = 0.5 Hz, lowpasscutoff = 40 Hz, and epochs length = 2.0 s for EEGNet4,2 pipeline.

After epoch extraction, all classifiers were trained using data from all offline runs. For the EEGNet4,2 pipeline, offline runs 1 to 5 were used for training, and run 6 was used for validation during model training. In contrast, all six offline runs were used for training in the other pipelines. Data from all online runs were used to evaluate model performance. Performance was assessed using both classification accuracy and ITR. The final output of the BCI system (i.e., the predicted letter or symbol) was determined by selecting the class with the highest mean classifier output, as was done in the online experiment (Section 2.5). For each sequence (from 1 to 15), classification was performed using cumulative data up to that sequence, and the corresponding accuracy and ITR were computed (e.g., for sequence 5, all data from sequences 1 to 5 were used for the target letter classification).

### 2.13 BCI simulation with dynamic stopping

The analysis method was identical to that described in Section 2.12, except for the following modification. Instead of evaluating performance after each sequence, Dynamic Stopping (DS) was applied (Verschore et al., 2012; Schreuder et al., 2011a, 2013).

In this analysis, the system first waited until a predefined minimum number of sequences had been presented. After this point, for each subsequent stimulus, a one-sided Welch's *t*-test was conducted between the classification score of the class with the highest mean output and those of the remaining classes. If a statistically significant difference was found at α = 0.05, the trial was terminated at that stimulus, and the system output was determined based on the data available up to that point. In this study, the minimum number of sequences was varied from 2 to 14 in steps of one.

## 3 Results

### 3.1 ERP analysis results

**Figure 3:**
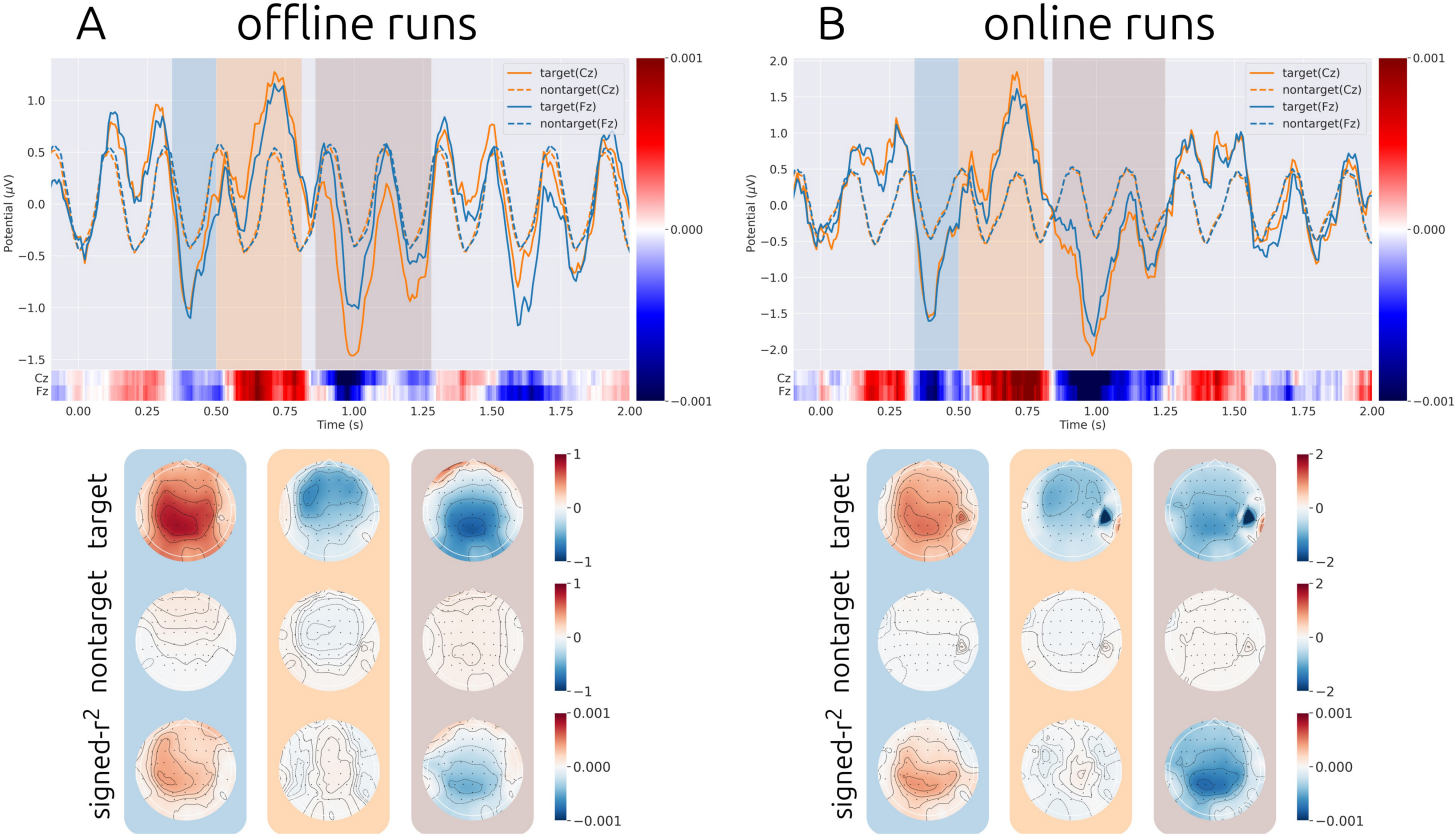
Grand average event-related potential (ERP) responses, excluding subject 10, who exhibited excessive noise. The orange and blue lines show the time course for channel Cz and Fz, respectively. The solid and dashed lines are responses to the target and non-target stimuli, respectively. The color bars below time course depicts signed-r^2^ values. The topography map shows the spatial distribution of responses to target, non-target stimuli, and signed-r^2^ over scalp, respectively. The blue, orange, and brown shaded regions in the ERP time courses correspond to the background colors of the topographical maps. These time windows correspond to the blue, orange, and brown shaded regions, which represent the N2, P300, and N700 components, respectively. **(A)** Results for data in offline runs. **(B)** Results for data in online runs. Distinct ERP responses were observed for target stimuli in both offline and online runs.

Figures 3A, B depict the grand-average ERP responses for the offline and online runs, respectively (see Supplementary Figure S1 for the average ERP responses for each participant). For both conditions, P300 components were observed around 0.5–0.8 s. A negative deflection around 0.3–0.5 s, predominantly over frontal areas, is considered to reflect the N2 component. Additionally, a late negative component (N700) was observed around 0.9–1.0 s. These three components (P300, N2, and N700) exhibited large signed-*r*^2^ values, suggesting their contributions to classification performance.

When comparing ERP time courses between online and offline runs, both showed similar temporal patterns. However, the amplitudes and signed-*r*^2^ values were generally larger and more pronounced in the online runs.

In response to non-target stimuli, sinusoidal wave-like patterns synchronized with the SOA (0.2 s) were observed. These can be interpreted as steady-state responses to the non-target stimuli, consistent with previous findings (Kojima and Kanoh, 2024b,c).

### 3.2 Online experiment results

Table 2 summarizes the classification results from the online runs during the experiment. Two participants achieved an accuracy of 1.0, five participants fell within the range of 0.8 to 0.95, and two participants scored between 0.6 and 0.7. For subject 10, classification failed across all trials, resulting in an accuracy of 0.0. The mean classification accuracy across all participants was 0.76 ± 0.28 (mean ± standard deviation), which improved to 0.84 ± 0.13 when excluding subject 10.

**Table 2:** The letter selection accuracy and ITR in the online runs.

Participant	Accuracy	ITR (bits/min)	CI (Accuracy)	CI (ITR)
1	0.87	2.44	0.62–0.96	1.39–2.97
2	0.93	2.79	0.70–0.99	1.70–3.14
3	1.00	3.24	0.80–1.00	2.11–3.24
4	0.87	2.44	0.62–0.96	1.39–2.97
5	0.60	1.32	0.36–0.80	0.56–2.13
6	0.67	1.57	0.42–0.85	0.72–2.35
7	1.00	3.24	0.80–1.00	2.10–3.24
8	0.87	2.44	0.62–0.96	1.39–2.97
9	0.80	2.12	0.55–0.93	1.14–2.77
10	0.00	0.03	0.00–0.20	0.03–0.20
Average	0.76 ± 0.28	2.16 ± 0.93	0.57–0.91	1.55–2.70
Average w/o 10	0.84 ± 0.13	2.40 ± 0.63	0.76–0.93	1.99–2.79

The classification task was 30-class letter selection. The statistical chance level was 0.13. For each subject, 95% confidence intervals (CIs) for accuracy were computed using the Wilson score interval, and the corresponding CIs for ITR were derived from the corresponding bounds of the accuracy CIs. The 95% CIs of the average accuracy and ITR were estimated using bootstrap resampling across participants (10,000 iterations). Average values are reported as mean ± standard deviation across all participants, while “Average w/o 10” indicates the mean ± standard deviation excluding subject 10.

**Figure 4:**
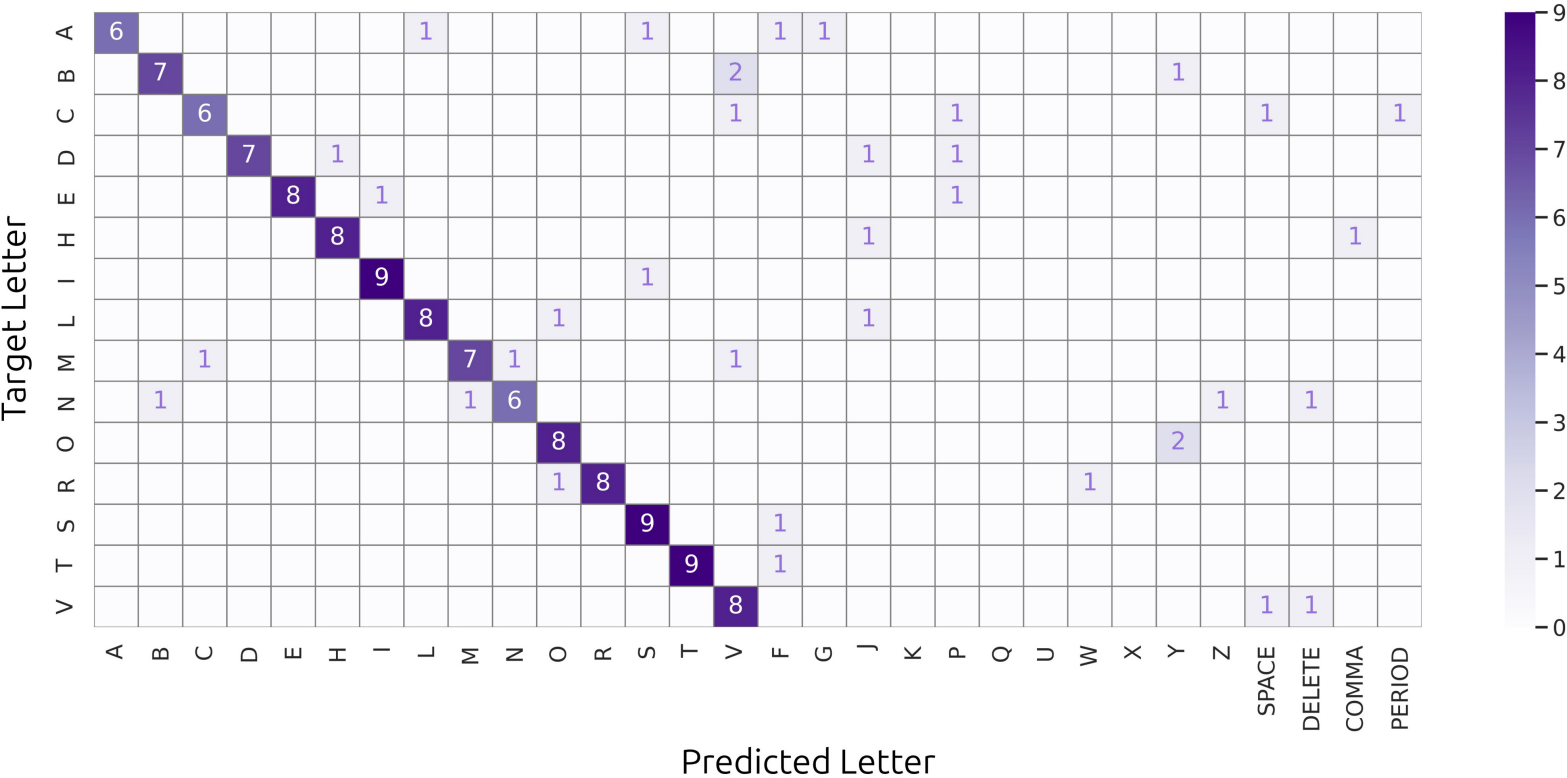
Confusion matrix summarizing the classification results in the online runs, aggregated across all participants. Rows and columns represent the target and predicted letters, respectively. Diagonal elements indicate correct classifications, while off-diagonal elements represent misclassifications. The color intensity reflects the number of trials for each target and prediction pair. The dominance of diagonal elements suggests that the classifier successfully distinguished target letters in most trials.

Figure 4 shows the confusion matrix summarizing the classification results from the online runs, aggregated across all participants. The dominance of the diagonal elements suggests that the classifier successfully distinguished the target letters in most trials.

Regarding the ITR, two participants achieved 3.24 bits/min, while five others obtained values between 2.0 and 3.0 bits/min. The overall mean ITR was 2.16 ± 0.93 bits/min, and 2.40 ± 0.63 bits/min when excluding subject 10.

### 3.3 Effect of artifact removal

The mean classification accuracy in the online experiment was 0.76 ± 0.28, whereas the ICA-based artifact removal simulation yielded 0.74 ± 0.29. A Wilcoxon signed-rank test conducted on the classification accuracy across the 10 participants revealed no statistically significant difference between the two conditions (*p* = 0.31).

### 3.4 Effect of class imbalance

Table 3 shows the simulation results obtained when varying the class ratio in the training data, as described in Section 2.9. For class ratios ranging from *N*_*T*_:*N*_*nT*_ = 1:29 to 1:5, the letter selection accuracy remained within the range of 0.72–0.78 across all conditions, with no substantial differences observed. In contrast, when the class ratio was set to 1:1, the accuracy decreased to 0.61.

**Table 3:** Classification accuracy under different class imbalance ratios.

Ratio (*N*_*T*_:*N*_*nT*_)	*N*_*T*_:*N*_*nT*_	Accuracy
1:1	450:450	0.61 ± 0.25
1:5	450:2, 250	0.72 ± 0.28
1:10	450:4, 500	0.75 ± 0.28
1:20	450:9, 000	0.76 ± 0.30
1:29	450:13, 050	0.78 ± 0.29

The ratio (*N*_*T*_:*N*_*nT*_) denotes the number of target and non-target samples used for training. Accuracy values are reported as mean ± standard deviation across participants.

### 3.5 Optimal preprocessing parameters and classification pipeline

Table 4 presents the preprocessing parameters that yielded the highest AUC scores for each classification pipeline. Among all tested parameter combinations, the LDA pipeline with a high-pass cutoff at 0.5 Hz, low-pass cutoff at 8 Hz, and epoch length at 2.0 s achieved the highest AUC score of 0.747. Except for EEGNet4,2, all pipelines performed best with the same combination of high-pass cutoff at 0.5 Hz, low-pass cutoff at 8 Hz, and epoch length at 2.0 s. In contrast, EEGNet4,2 achieved the best AUC when the high-pass cutoff was 0.5 Hz, the low-pass cutoff was 40 Hz, and epoch length was 2.0 s, suggesting that EEGNet4,2 benefits from input data with a broader frequency band compared to the other pipelines.

**Table 4:** Preprocessing parameters that yielded the highest AUC for each classification pipeline, along with the corresponding AUC values.

Pipeline	Highpass cutoff (Hz)	Lowpass cutoff (Hz)	Epochs Tmax (s)	AUC
LDA	0.5	8	2.0	0.747
xdw+LDA	0.5	8	2.0	0.736
xdwCov+TS+LDA	0.5	8	2.0	0.717
EEGNet4,2	0.5	40	2.0	0.741

The high-pass and low-pass cutoff frequencies define the band-pass filter applied during preprocessing. Epoch length (Tmax) denotes the duration of EEG segments extracted relative to stimulus onset. The optimal parameters were selected based on cross-validation performance on the offline data.

### 3.6 BCI performance with early stopping

**Figure 5:**
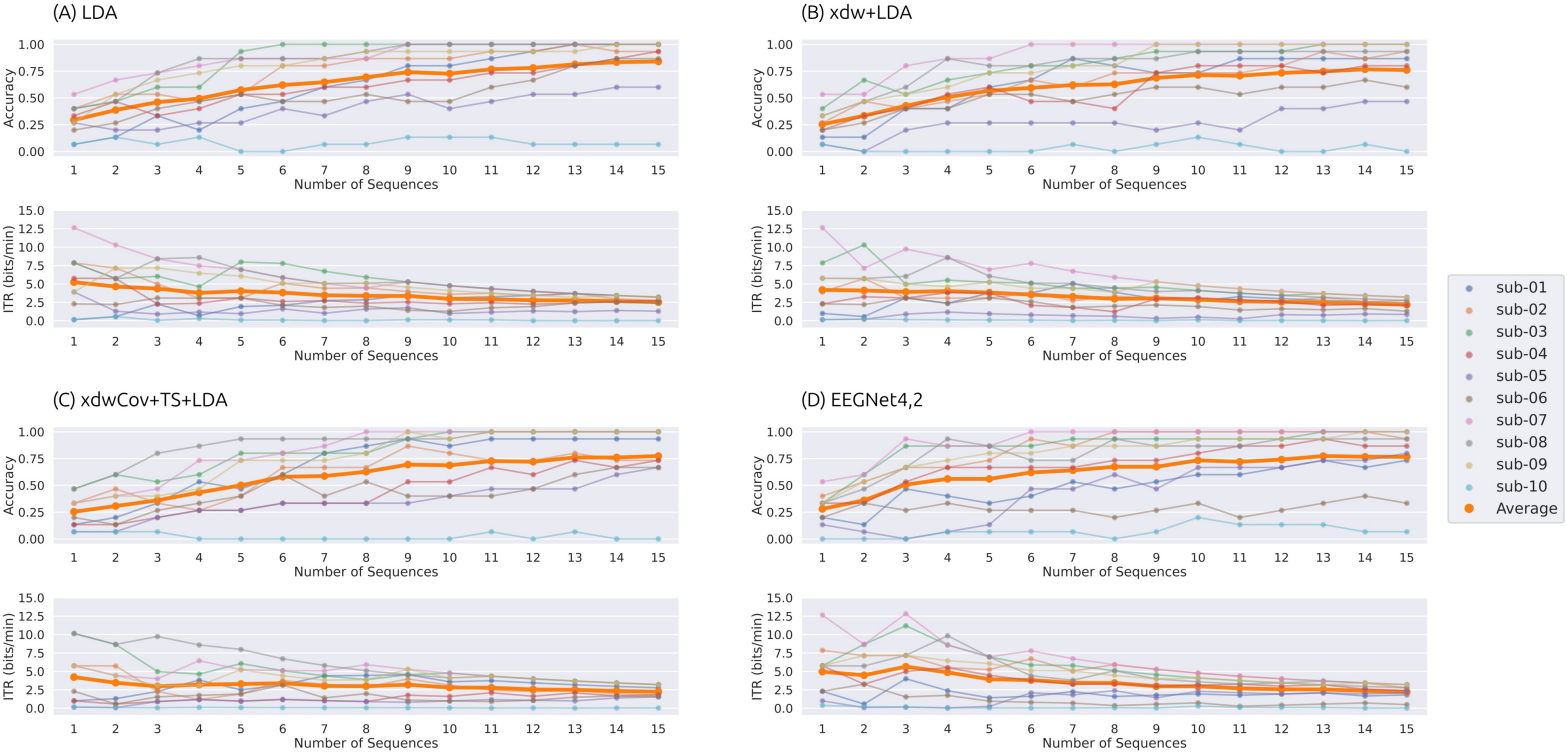
Classification accuracy and information transfer rate (ITR) as a function of the number of sequences under the static stopping condition. Results are shown for four classification pipelines: **(A)** LDA, **(B)** xdw+LDA, **(C)** xdwCov+TS+LDA, and **(D)** EEGNet4,2. The top panels show classification accuracy, and the bottom panels show ITR (bits/min). Thin lines represent individual participants, and thick orange lines indicate the average across participants.

**Figure 6:**
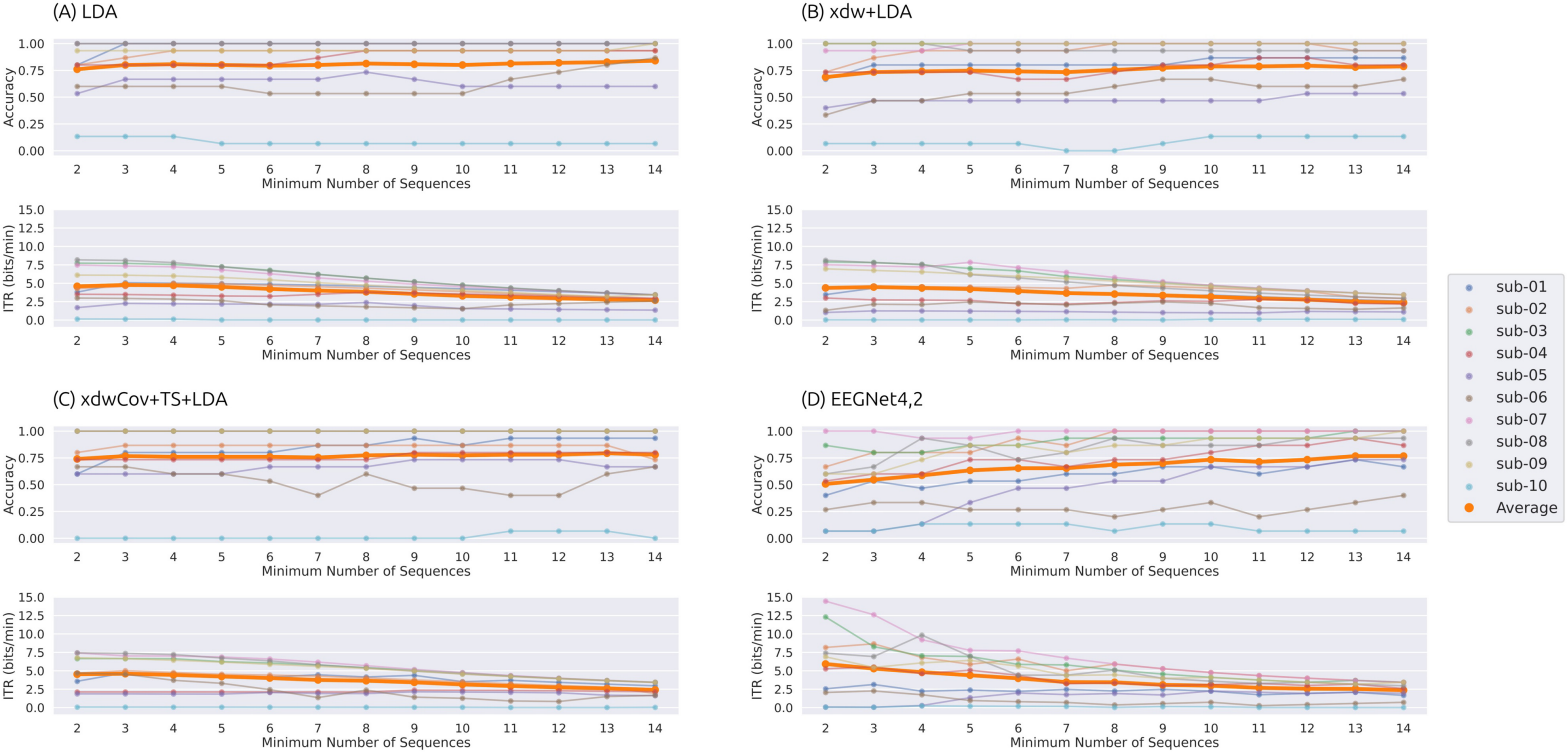
Classification accuracy and information transfer rate (ITR) as a function of the minimum number of sequences under the dynamic stopping condition. Results are shown for four classification pipelines: **(A)** LDA, **(B)** xdw+LDA, **(C)** xdwCov+TS+LDA, and **(D)** EEGNet4,2. The top panels show classification accuracy, and the bottom panels show ITR (bits/min). Thin lines represent individual participants, and thick orange lines indicate the average across participants. Dynamic stopping was triggered when a statistically significant difference was detected after exceeding the minimum number of sequences.

Figures 5, 6 show the classification accuracy and ITR achieved with static stopping and dynamic stopping, respectively. Table 5 summarizes the optimal number of sequences for static and dynamic stopping, along with the corresponding accuracy and ITR values. The optimal number of sequences was determined under the following three criteria: (1) maximizing accuracy, (2) maximizing ITR, and (3) maximizing ITR while maintaining accuracy ≥0.7. An accuracy of 0.7 has been reported to enable effective communication in BCI-based communication systems (Kleih et al., 2015; Kübler et al., 2001).

**Table 5:** Summary of classification performance under static and dynamic stopping strategies across four classification pipelines.

Stopping strategy	Maximized metrics	Pipeline	NS	ST	Acc.	ITR
Static stopping	Accuracy	LDA	15	91.8	0.840 ± 0.28	2.53 ± 1.0
		xdw+LDA	14	85.8	0.767 ± 0.28	2.33 ± 1.1
		xdwCov+TS+LDA	15	91.8	0.773 ± 0.29	2.23 ± 1.0
		EEGNet4,2	13	79.8	0.773 ± 0.29	2.54 ± 1.2
	ITR	LDA	1	7.8	0.293 ± 0.14	5.24 ± 3.7
		xdw+LDA	1	7.8	0.253 ± 0.14	4.18 ± 3.7
		xdwCov+TS+LDA	1	7.8	0.253 ± 0.15	4.22 ± 3.7
		EEGNet4,2	3	19.8	0.507 ± 0.31	5.64 ± 4.1
	ITR (Acc. ≥0.70)	LDA	9	55.8	0.740 ± 0.27	3.37 ± 1.7
		xdw+LDA	10	61.8	0.713 ± 0.29	2.88 ± 1.6
		xdwCov+TS+LDA	11	67.8	0.727 ± 0.31	2.78 ± 1.6
		EEGNet4,2	10	61.8	0.733 ± 0.27	2.97 ± 1.5
Dynamic stopping	Accuracy	LDA	14	86.7 ± 1.5	0.840 ± 0.28	2.70 ± 1.1
		xdw+LDA	12	77.6 ± 4.0	0.793 ± 0.27	2.76 ± 1.3
		xdwCov+TS+LDA	13	81.8 ± 2.8	0.793 ± 0.28	2.60 ± 1.2
		EEGNet4,2	13	80.2 ± 1.2	0.767 ± 0.30	2.53 ± 1.3
	ITR	LDA	3	48.5 ± 9.8	0.800 ± 0.26	4.76 ± 2.5
		xdw+LDA	3	45.6 ± 9.5	0.733 ± 0.29	4.48 ± 2.7
		xdwCov+TS+LDA	3	47.9 ± 12.9	0.767 ± 0.29	4.60 ± 2.4
		EEGNet4,2	2	18.3 ± 2.6	0.507 ± 0.30	5.92 ± 4.7
	ITR (Acc. ≥0.70)	LDA	3	48.5 ± 9.8	0.800 ± 0.26	4.76 ± 2.5
		xdw+LDA	3	45.6 ± 9.5	0.733 ± 0.29	4.48 ± 2.7
		xdwCov+TS+LDA	3	47.9 ± 12.9	0.767 ± 0.29	4.60 ± 2.4
		EEGNet4,2	9	56.6 ± 1.4	0.700 ± 0.29	3.09 ± 1.8

For each stopping method and classification pipeline, the table shows the number of sequences (static stopping) or the minimum number of sequences (dynamic stopping) at which each performance metric was maximized. Three metrics were considered: Accuracy, ITR (information transfer rate), and ITR with a constraint that Accuracy ≥0.70. All reported values are averaged across 10 participants. Values reported with ± are expressed as mean ± standard deviation across participants. NS, number of sequences; ST, selection time (s); Acc., accuracy; ITR, bits/min.

**Static stopping:** When static stopping was applied and accuracy was maximized, the best accuracy was achieved by using all 15 sequences. When ITR was maximized, EEGNet4,2 yielded the highest ITR and accuracy. LDA achieved the second-highest ITR, but its accuracy was significantly lower than that of EEGNet4,2. When optimizing ITR while maintaining accuracy ≥0.7, LDA and EEGNet4,2 showed comparable ITR and accuracy values.

**Dynamic stopping:** When dynamic stopping was applied and accuracy was maximized, LDA achieved the highest accuracy by triggering dynamic stopping after 14 sequences, while EEGNet4,2 showed the lowest accuracy. When ITR was maximized, EEGNet4,2 yielded the highest ITR. When ITR was maximized under the constraint of accuracy ≥0.7, LDA achieved the highest ITR. Furthermore, for EEGNet4,2, applying dynamic stopping after two sequences resulted in the highest performance observed in this study for subject 7 (accuracy of 1.0, ITR of 14.44 bits/min, and an average selection time of 20.4 s), while subject 3 achieved an accuracy of 0.87, an ITR of 12.30 bits/min, and an average selection time of 18.0 s.

Overall, the highest accuracy (0.840) and an ITR of 2.70 bits/min were obtained using the LDA pipeline with dynamic stopping triggered after 14 sequences. The highest ITR (5.92 bits/min) was obtained using EEGNet4,2 with dynamic stopping triggered after 2 sequences, although the corresponding accuracy was relatively low (0.507). Under the constraint of maintaining accuracy ≥0.7, the optimal configuration was LDA with dynamic stopping triggered after 3 sequences (average selection time of 48.5 s), yielding an accuracy of 0.800 and an ITR of 4.76 bits/min.

## 4 Discussion

### 4.1 ERP responses

From the ERP analysis, the N2, P300, and N700 components were observed exclusively in response to target stimuli. The signed-r^2^ values indicated high separability between responses to target and non-target stimuli, suggesting that distinct ERP responses were reliably elicited during the ASME-speller task.

When comparing ERP time courses between offline and online runs, the responses in the online runs exhibited larger amplitudes. One possible explanation is that participants had become more familiar with the ASME-speller task during the online phase, as the offline runs were conducted earlier in the session. Increased task familiarity may have contributed to the enhanced ERP amplitudes. Another possible explanation is that the feedback provided during the online runs, which was absent in the offline runs, may have increased participants' motivation. Previous studies have shown that increased motivation enhances the amplitude of the P300 component (Kleih et al., 2010).

The latency of ERP components was longer than those typically observed in standard auditory oddball tasks. This is likely due to the use of voice stimuli. Each auditory stimulus consisted of a spoken alphabet letter; however, the onset of the spoken audio did not precisely align with the stimulus onset, and the exact moment when the participant recognized the letter might vary. These factors likely contributed to the delayed ERP latencies observed in this study.

### 4.2 Online experiment

As shown in Table 2, seven out of ten participants achieved an accuracy greater than 0.70, which is reported to enable effective communication (Kleih et al., 2010; Kübler et al., 2001). The mean accuracy across all participants was 0.76. Considering the theoretical chance level (1/30 = 0.033), most participants performed well above chance. Furthermore, excluding subject 10, all participants achieved accuracies significantly above the statistical chance level (0.13 at *p* = 0.05 Combrisson and Jerbi, 2015). When considering the 95% confidence intervals (CIs) of accuracy, the lower bounds exceeded the statistical chance level for all participants except subject 10, further confirming that the proposed system achieved performance significantly above chance for most participants. Furthermore, for three participants, the lower bounds of the CIs exceeded 0.70. In addition, the lower bound of the CI for the mean accuracy excluding subject 10 also exceeded 0.70. These results suggest that, for some participants, the system can reliably achieve the accuracy level required for effective communication in BCI applications, even when uncertainty in the accuracy estimates is taken into account.

Subject 10 exhibited an accuracy of 0.0, which was likely due to excessive body movement during the measurement. This result highlights the critical impact of EEG recording quality on BCI performance. However, in the *post-hoc* analysis, the performance for this participant improved up to 0.20, which is above the statistical chance level mentioned above, indicating that appropriate signal processing and machine learning techniques have the potential to improve performance even under suboptimal recording conditions.

Although artifact removal was not applied in the online experiment, the results presented in Section 3.3 showed that removing EOG artifacts did not result in a significant difference in classification accuracy. These findings suggest that the impact of EOG artifacts on the experimental data was limited.

### 4.3 Effect of class imbalance

In the design of the present study, a class imbalance of 1:29 was present, raising concerns that this imbalance might affect classification performance and the learning process of LDA. However, as shown in Table 3, the results indicate that classification accuracy decreased as the class ratio *N*_*T*_:*N*_*nT*_ approached 1:1. This result is likely attributable to the reduced number of non-target samples, which may degrade the estimation accuracy of the non-target class mean vector and the within-class covariance matrix. In particular, in the present paradigm, the SOA was set to 0.2 s, which is relatively short. As a result, responses to non-target stimuli adjacent to target stimuli may be observed as temporally shifted waveforms that are similar to target responses. Since such non-target responses share characteristics with target responses, learning their distribution sufficiently is likely important for improving classification performance.

These findings suggest that, in the present framework, using all available data including a sufficient number of non-target samples is more important for stable classification performance than simply balancing the class ratio.

### 4.4 Optimal preprocessing parameters

From Table 4, it was revealed that the optimal EEG epoch length was longer than that used in the online experiment (2.0 s vs. 1.0 s). This is supported by the average ERP responses (Figure 3), where informative ERP components were observed beyond 1.0 s.

Regarding EEG filtering parameters, the optimal high-pass cutoff was 0.5 Hz across all pipelines. This result aligns with previous findings (Qassim et al., 2013), which reported that ERP signals predominantly occupy the lower frequency range (0.5–8.0 Hz). For the low-pass cutoff frequency, 40 Hz was optimal for EEGNet4,2, while 8 Hz was optimal for the other pipelines. This difference may be attributed to the first convolutional layer of EEGNet4,2 acting as a filter (Lawhern et al., 2018), allowing the model to learn the optimal filtering parameters internally.

When comparing classification pipelines, LDA exhibited the highest AUC, followed by EEGNet4,2. Although xdw+LDA and xdwCov+TS+LDA yielded slightly lower AUC scores, their performance was comparable to each other.

### 4.5 Early stopping procedures

We compared two early stopping procedures: static stopping and dynamic stopping. As shown in Table 5, dynamic stopping yielded the highest scores for both accuracy and ITR.

In particular, when using LDA and maximizing accuracy, both static and dynamic stopping achieved identical accuracies; however, ITR was higher with dynamic stopping. This is because static stopping waits for the completion of all 15 sequences, whereas dynamic stopping begins evaluating classifier outputs after the 14th sequence and determines the final output on a stimulus-by-stimulus basis. As a result, some trials can be terminated earlier within the 15th sequence, reducing trial duration and improving ITR.

As shown in Figures 5, 6, accuracy fluctuated more with static stopping as the number of sequences varied, whereas it remained relatively stable with dynamic stopping. Additionally, Table 5 shows that dynamic stopping generally achieved higher peak performance than static stopping. Notably, when maximizing ITR under the constraint of accuracy ≥0.7, dynamic stopping outperformed static stopping in both ITR and accuracy for all the pipelines. These findings suggest that, for the ASME-speller task, dynamic stopping is more robust and better suited in terms of both accuracy and information transfer rate.

We further investigated the effect of the minimum number of sequences for dynamic stopping based on the results in Figure 6. For LDA, xdw+LDA, and xdwCov+TS+LDA, changes in the minimum number of sequences had little impact on accuracy or ITR. Even with a minimum as low as 3 sequences, accuracy remained around 0.75. In contrast, EEGNet4,2 exhibited significant variability in both accuracy and ITR depending on the minimum number of sequences. In particular, subjects 3 and 7 showed substantial variability in ITR. These participants achieved high accuracies exceeding 0.85 even when the minimum number of sequences was set to 2. Therefore, delaying the triggering of dynamic stopping likely led to unnecessarily prolonged trial durations, resulting in a reduction in ITR. These findings suggest that user-specific tuning of the minimum number of sequences is necessary to achieve optimal performance.

### 4.6 Comparison between pipelines

In this study, we evaluated four classification pipelines: (1) LDA, (2) xdw+LDA, (3) xdwCov+TS+LDA, and (4) EEGNet4,2.

Surprisingly, the LDA pipeline exhibited the highest accuracy across most conditions. This pipeline has been employed in our previous studies (Kojima and Kanoh, 2024b,c; Kojima et al., 2025), and LDA is widely used in BCI research (Lotte et al., 2018). Our results reaffirm the robustness and reliability of the well-established LDA approach.

The findings suggest that the optimal pipeline depends on whether the goal is to maximize accuracy, ITR, or achieve a balance between the two. When prioritizing accuracy, LDA was shown to be the most suitable option. When prioritizing ITR, EEGNet4,2 combined with dynamic stopping achieved the highest performance, recording 5.92 bits/min. Although its accuracy was 0.507, this may still be considered acceptable given the large number of classes. Moreover, since the current task is a speller, occasional classification errors may still permit effective communication, indicating that this performance level could be practically acceptable in some cases. When balancing both accuracy and ITR, LDA with dynamic stopping was found to be the most appropriate, achieving high ITR while maintaining an accuracy above 0.7.

As shown in Figure 6D, when dynamic stopping was applied to EEGNet4,2, subjects 3 and 7 achieved ITRs of 12.30 and 14.44 bits/min, respectively, with accuracies of 0.86 and 1.0 at a minimum sequence number of 2. In contrast, under the same conditions, LDA achieved ITRs of 7.72 and 7.47 bits/min, both with accuracies of 1.0. Notably, these two participants also achieved perfect accuracy in the online experiment, suggesting that they exhibited highly separable ERP responses. For such users, using EEGNet4,2 with dynamic stopping may yield substantial improvements in ITR without compromising accuracy.

EEGNet4,2 showed larger inter-participant variability compared to the other pipelines. To investigate the factors underlying this variability, additional analyses were conducted (see Supplementary Section 1 for details). The results indicated that EEGNet4,2 exhibited lower training performance and generalization performance than LDA, as reflected in the AUC analysis, suggesting that the model may not have sufficiently fit the training data. On the other hand, very high performance was observed for some participants (e.g., subjects 3 and 7), indicating strong user-dependent effects. Furthermore, in EEGNet4,2, part of the data was reserved for validation, resulting in fewer samples being used for parameter updates compared to the other pipelines. This limitation in training data, combined with the model's sensitivity to participant-specific EEG characteristics, may have contributed to the observed variability. These findings suggest that user-specific optimization is important for deep learning-based pipelines.

Moreover, the performance of each pipeline may also be influenced by factors such as feature extraction methods, the number of xDAWN components, and classifier hyperparameters. These aspects should be further investigated in future work.

Overall, the results suggest that while LDA is the most robust and generally applicable pipeline, EEGNet4,2 may offer superior performance for certain individuals when appropriately optimized. Furthermore, the application of dynamic stopping enabled substantial improvements in ITR under many conditions while maintaining comparable levels of accuracy.

### 4.7 Comparing with previous auditory speller studies

Table 6 summarizes previous auditory BCI speller studies in terms of the number of classes, classification accuracy, information transfer rate (ITR), and selection time per letter.

**Table 6:** Summary of performance reported in previous auditory BCI speller studies.

Authors	Modality	NC	ITR	Acc.	ST
(Furdea et al. 2009)	Auditory	25	1.54	0.65	97.5
(Klobassa et al. 2009)	Auditory	36	1	0.55	98.0
(Höhne et al. 2011)	Auditory	9 (27)	3.4 (4.23)	0.89	31
(Schreuder et al. 2011b)	Auditory	36	2.84 & 5.26	0.77 & 0.86	DS
(Lu et al. 2019)	Auditory+visual	36	20	0.8	NR
(Klobassa et al. 2009)	Auditory+visual	36	2	0.75	98.0
(Zhang et al. 2020)	Auditory+tactile	36	11.66	0.73	ST
The present study (Online Results)	Auditory	30	2.16	0.76	90.8
The present study (LDA with DS)	Auditory	30	4.76	0.80	DS (48.5)

The table summarizes the number of classes (NC), information transfer rate (ITR, bits/min), accuracy (Acc.), and selection time (ST, s) for each study. The ITR values of (Höhne and Tangermann 2014) were estimated from figures in the original paper. For (Klobassa et al. 2009) and (Lu et al. 2019), both ITR and accuracy were extracted from figures. For (Lu et al. 2019), the reported ITR corresponds to the condition with a superposition time of 2 s. For (Schreuder et al. 2011b), the two values correspond to session 1 and session 2, respectively. For (Höhne et al. 2011), results are shown for the 9-class condition, with values in parentheses indicating performance when using the predictive text entry system (effectively 27 classes). NR indicates that the value was not reported, DS indicates dynamic stopping, and ST denotes static stopping where the optimal sequence length was determined in advance using training data. For the present study, the reported values correspond to (i) the online experiment and (ii) the results obtained using dynamic stopping (DS) with the LDA pipeline.

**Comparison with auditory-only BCI spellers:** First, we compare auditory-only BCI spellers. In terms of the number of selectable classes, many previous studies target approximately 30 classes. This is likely because a practical speller requires around 30 classes to cover the 26 alphabet letters along with several symbols or numbers. However, some studies do not support the full alphabet. For example, Furdea et al. (2009) and Markovinović et al. (2022) are limited to 25 and 22 classes, respectively, and therefore do not enable complete alphabet input.

In addition, many existing studies adopt multi-step selection strategies, in which a single letter is selected through multiple sequential decisions. For instance, row-column paradigms such as Furdea et al. (2009) and Klobassa et al. (2009) determine a letter in two steps by selecting its row and column. In these approaches, each step is formulated as a classification problem with approximately 5–6 classes. Similarly, Schreuder et al. (2011b) and Zhang et al. (2020) employ two-step selection with 6 classes per step to achieve a 36-class system. Thus, although multi-step approaches enable a large number of selectable letters, they require multiple selections per letter and often involve less intuitive mappings between auditory stimuli and target letters at each step.

In contrast, the proposed method and Höhne and Tangermann (2014) adopt a single-step strategy, in which all candidate letters are handled within a single multiclass classification. This single-step design enables more direct letter selection. On the other hand, Markovinović et al. (2022) allows 22-letter input in a single step; however, it further introduces a second step in which a subset of candidate letters is first estimated and then refined to determine the final output. Among systems that enable approximately 30-class input in a single step, the CharStreamer paradigm (Höhne and Tangermann, 2014) is the most similar to the proposed method. Note that Höhne et al. (2011) achieves 27-letter input by combining a 9-class classification paradigm with a predictive text entry system. However, since this framework differs from a standard multiclass classification setting, direct comparison with the present study is not straightforward. Therefore, unless otherwise noted, this study is excluded from the following comparisons.

Overall, auditory-only BCI spellers exhibit diverse design strategies in terms of system architecture (single-step vs. multi-step) and the number of selectable classes. Within this landscape, the proposed method is characterized by enabling single-step letter selection while supporting 30 classes. In particular, compared with other single-step multiclass systems, such as CharStreamer (Höhne and Tangermann, 2014), the proposed method handles a full set of alphabet letters within a single classification framework.

Next, we compare classification accuracy. Previous studies have reported that effective communication using a BCI becomes feasible when the accuracy exceeds 0.7 (Kleih et al., 2015; Kübler et al., 2001). As shown in Table 6, among auditory-only BCI spellers, only (Schreuder et al., 2011b; Kleih et al., 2015) reported mean accuracies exceeding 0.7. It should be noted that Höhne et al. (2011) reported a high accuracy of 0.89; however, this value corresponds to a 9-class classification task and is not directly comparable to final letter selection accuracy.

In the present study, the mean accuracy exceeded 0.7 both in the online experiment and when combining LDA with dynamic stopping. This suggests that the proposed method can achieve the level of accuracy required for effective communication. Furthermore, compared with the CharStreamer paradigm (Höhne and Tangermann, 2014), which also adopts a single-step multiclass approach, the reported mean letter selection accuracy was 0.35, whereas the proposed method achieved substantially higher accuracy.

Next, we compare selection time. In studies employing fixed-length selection protocols (Furdea et al., 2009; Klobassa et al., 2009; Markovinović et al., 2022), the selection time per letter is typically around 95–110 s. However, Furdea et al. (2009) and Klobassa et al. (2009) adopt two-step input paradigms; therefore, the processing time per step is approximately half of the total selection time. Consequently, direct comparison with these methods should be interpreted with caution.

In the present study, the average selection time in the online experiment was 90.8 s, which is on a similar time scale to existing studies using fixed-length protocols. It should be noted that studies employing dynamic or static stopping often do not explicitly report average selection time, making strict comparisons difficult. Therefore, in this section, we primarily compare with studies using fixed-length settings. On the other hand, when dynamic stopping was applied with LDA in the present study, the average selection time was reduced to 48.5 s. This result suggests that selection time can be substantially shortened while maintaining classification performance.

Finally, we compare the ITR. As defined in Equations 1, 2, ITR is a function of the number of selectable classes, classification accuracy, and selection time per letter. Therefore, it reflects the combined influence of these factors. In particular, a trade-off is expected between selection time and accuracy. Reducing the selection time decreases the number of samples available for classification, which may lead to a reduction in accuracy. Since the number of classes is largely constrained by system design, improving ITR requires reducing selection time without substantially degrading accuracy. For this reason, methods employing static or dynamic stopping tend to achieve higher ITR. For example, Zhang et al. (2020) reported a high ITR of 8.63 bits/min using static stopping. However, the corresponding accuracy was 0.64, which is below the threshold of 0.7 often considered necessary for effective communication. When restricting the comparison to conditions with accuracy above 0.7, Schreuder et al. (2011b) reported high performance, achieving an ITR of 2.84 bits/min with an accuracy of 0.77. In addition, in a second session including only participants who achieved sufficient performance in the first session, an ITR of 5.26 bits/min with an accuracy of 0.86 was obtained. This system also supports 36 classes, demonstrating strong performance as an auditory BCI speller. However, it requires a setup with six loudspeakers, which increases hardware complexity. Furthermore, Höhne et al. (2011) reported an ITR of 4.23 bits/min for 27-letter selection. However, the ITR for the 9-class classification alone was 3.4 bits/min, suggesting that the additional improvement (0.83 bits/min) was due to the predictive text entry system. This indicates that auxiliary techniques such as predictive text entry can contribute to improving ITR in spelling applications.

In the present study, an ITR of 2.16 bits/min was achieved in the online experiment, which increased to 4.76 bits/min when combining LDA with dynamic stopping. Compared with Zhang et al. (2020), the ITR is lower; however, when considering the auditory-only condition reported in that study (i.e., without tactile stimulation), the corresponding accuracy was lower than that of the present study. Compared with Schreuder et al. (2011b), the performance of the proposed method lies between the results reported for the first and second sessions. Considering that the second session included only high-performing participants, the proposed method may achieve performance comparable to that study.

Moreover, compared with CharStreamer (Höhne and Tangermann, 2014), which also adopts a single-step multiclass approach, the proposed method achieves substantially higher ITR [1.3 bits/min vs. 2.16 bits/min (online) and 4.76 bits/min (LDA + dynamic stopping)]. Overall, the proposed method achieves a comparable ITR while maintaining a balance between accuracy and selection time, and while enabling single-step multiclass input.

**Comparison with hybrid BCI spellers:** We compare the proposed method with multimodal auditory BCI systems. Zhang et al. (2020) reported a 36-class system combining auditory and tactile stimuli, achieving an accuracy of 0.73 and an ITR of 11.66 bits/min. In the same study, both an auditory-only system and a multimodal system with tactile stimuli were evaluated, and the addition of tactile stimulation improved the accuracy by 0.09 and the ITR by 3.0.

As for systems combining visual stimuli, Lu et al. (2019) reported an accuracy of 0.80 and an ITR of 20 bits/min for a 36-class system, which is the highest performance among the studies considered in this work. Furthermore, Klobassa et al. (2009) also showed that combining auditory and visual stimuli improved both accuracy and ITR compared to an auditory-only system.

On the other hand, these multimodal systems require additional devices, such as tactile stimulation equipment, which increases the complexity of the system. In addition, systems that rely on visual stimuli may not be suitable for users with visual impairments.

Nevertheless, since higher performance has consistently been reported in multimodal systems compared to auditory-only systems, incorporating hybrid approaches, such as combining the ASME-speller with rapid serial visual presentation (RSVP) paradigms (Lees et al., 2018), may be effective for improving performance, and this remains an important direction for future work.

In summary, although the performance of the present study does not reach that of hybrid systems incorporating visual or tactile stimuli, it demonstrates sufficient performance among systems based solely on auditory stimuli that cover the full set of alphabet letters. In addition, many previous studies require two-step selections, employ stimulus-letter mappings that are not intuitive, or require additional physical devices such as multiple loudspeakers, displays, or tactile stimulation equipment. In contrast, the ASME-speller can be used with only widely available headphones and enables letter input in a single step through an intuitive stimulus sequence. Therefore, the proposed system has the potential to serve as a practical communication tool.

### 4.8 Toward use by patients with motor disabilities

Previous surveys of BCI needs in patients with severe neurological disorders have highlighted the importance of direct communication tools (Branco et al., 2023). Auditory BCI spellers are particularly promising in this context because they can be used by individuals who have difficulty with gaze control (Choi et al., 2023).

However, several challenges remain for the application of auditory BCIs in clinical populations. For example, Séguin et al. (2024) evaluated a two-class auditory BCI in patients with severe motor disabilities and reported that only a minority of participants achieved performance above chance level.

The present study evaluated the proposed ASME-speller only with healthy participants, and its usability for patients remains to be investigated in future work. Previous studies have shown that user training for patients can substantially improve auditory BCI performance (Simon et al., 2015; Halder et al., 2016). In particular, (Halder et al. 2016) reported that training increased the ITR by up to 18 times. Therefore, developing effective training protocols will be an important step toward enabling practical use of the ASME-speller for patients.

In addition, several techniques may further improve performance in patient populations. For example, Spüler et al. (2012) demonstrated that incorporating error-related potentials can improve the performance of a visual P300 speller in ALS patients. Moreover, Tou et al. (2026) showed that individual optimization of EEG electrode sets can improve BCI performance. Evaluating such approaches in the ASME-speller will be an important direction for future work.

### 4.9 Limitations and future work

Although the ASME-speller demonstrated promising performance in terms of classification accuracy and ITR, several limitations remain and should be addressed in future studies.

First, all participants in the current study were healthy, young, Japanese-speaking adults. To assess the broader applicability and usability of the system, future work should include more diverse participants, including older adults and non-Japanese speakers. Furthermore, evaluating the ASME-speller with individuals with neurological disorders would be beneficial to assess its potential as a practical assistive communication technology for this population.

The current system utilizes a 64-channel EEG setup, which may not be practical for real-world deployment. Evaluating whether similar BCI performance can be achieved using more affordable and portable EEG systems with fewer channels (e.g., 16 or 8 channels) is essential for realizing practical applications.

While 30 voice stimuli were used, it remains unclear whether each stimulus consistently elicits similar ERP responses. A systematic analysis of ERP waveforms across different stimuli is beneficial. Furthermore, developing a stimulus set that evokes consistent and comparable ERPs across all letters, regardless of which letter is attended, could enhance system robustness and classification performance.

Although several classification pipelines and preprocessing configurations were examined in the *post-hoc* analyses, there is still room to explore a broader range of classifiers, feature extraction methods, and their associated hyperparameters. Future work should investigate these additional pipelines to identify more optimal configurations.

The current evaluation of early stopping was performed using all offline data for training and assessed using online data, which was suitable for estimating the upper bound of performance. However, in realistic BCI use cases, such optimizations must be performed using only training data recorded during offline sessions. Future research should examine how well early stopping strategies perform when optimized under such constraints.

In the online experiment, a fixed subset of 15 letters was tested for all participants from the full set of 30 letters. In the proposed system, ERP detection is performed using a binary classifier that estimates whether an ERP response is elicited by each presented stimulus. The final output letter is determined based on the classifier responses to all stimuli presented within a trial. Therefore, if similar ERP responses can be elicited for all target stimuli, it is expected that any letter could be correctly identified. However, this assumption was not systematically evaluated in the present study and remains an important topic for future work.

Auditory BCIs have been reported to impose a higher workload than visual BCIs (An et al., 2014). In addition, the trial duration in the present study was relatively long (approximately 90 s). In the proposed ASME-speller, users are required to attend both to the relevant auditory stream and to the target letter within that stream, which may impose additional cognitive demands. In our previous study (Kojima and Kanoh, 2024b), we evaluated the workload of a four-class ASME task using the NASA-TLX (Hart and Staveland, 1988; Hart, 2006). The results showed that even when the number of classes and the trial duration were identical, the workload differed depending on the stimulus presentation method. This suggests that the workload in the ASME-speller could potentially be reduced by optimizing the stimulus presentation method. Furthermore, natural stimuli such as the speech stimuli used in this study have been reported to provide higher usability than classical beep-based stimuli (Höhne et al., 2012; Huang et al., 2018). However, the workload and usability of the proposed system were not evaluated in this study, as the primary objective of this work was to demonstrate the proof-of-concept feasibility of the ASME-speller. Future work should include systematic assessment of cognitive load and usability using standardized measures such as NASA-TLX, as well as direct within-participant comparisons with existing auditory BCI spellers, and analyses of the relationship between workload and BCI performance.

Finally, the current study did not incorporate participant-specific parameter tuning. Given the observed variability in classification accuracy across participants, personalizing preprocessing and classification settings may further improve performance and system robustness.

Addressing these limitations will be essential for enhancing the usability, generalizability, and practical deployment of the ASME-speller in real-world BCI applications.

## 5 Conclusion

In this study, we investigated the ASME-speller, a 30-class auditory BCI speller system that leverages auditory stream segregation. The ASME-speller presents stimuli according to the QWERTY keyboard layout, allowing users to selectively attend to the desired letter in the corresponding auditory stream. In this system, spoken alphabet stimuli corresponding to each row of the QWERTY layout are presented within multiple perceptually segregated auditory streams. Through this design, the user first restricts their attentional focus to a subset of letters by selectively attending to a specific auditory stream, and subsequently selects the desired letter by directing attention to the target stimulus within that stream. Furthermore, because the vertical arrangement of rows in the QWERTY layout is associated with the pitch of the auditory streams, the user can intuitively direct attention to the target stream.

In an EEG experiment with ten participants, ERP components such as P300, N2, and N700 were observed exclusively in response to target stimuli. During the online runs conducted after the offline training sessions, the system achieved an average accuracy of 0.76 and an average ITR of 2.16 bits/min. Excluding subject 10, who failed to achieve control, the averages increased to 0.84 and 2.40 bits/min, respectively. These values are well above both the theoretical chance level (1/30 = 0.033) and the statistical chance level (0.133), supporting the feasibility of the ASME-speller as an online BCI speller system.

Furthermore, we conducted *post-hoc* analyses to explore preprocessing parameters, classification pipelines, and early stopping strategies suitable for ERP classification in this task. The results showed that the LDA pipeline combined with dynamic stopping yielded robust performance across participants, achieving both high accuracy and ITR. For the best participant, EEGNet4,2 combined with dynamic stopping achieved an ITR of 14.44 bits/min and an accuracy of 1.0. Importantly, when compared with previous auditory BCI speller studies, the ASME-speller achieved ITR values within the range of previously reported systems and demonstrated performance comparable to existing approaches, while requiring only a simple headphone setup. These findings highlight the practicality of the ASME-speller as an auditory BCI speller system.

## Data Availability

All relevant data are publicly available on the Harvard Dataverse repository (https://doi.org/10.7910/DVN/TYRCWL).
