# Supplementary material for: The ASME-speller: 30-class auditory brain-computer interface speller using stream segregation and the QWERTY layout
Source: Front Hum Neurosci. 2026 May 21;20:1807535. doi: 10.3389/fnhum.2026.1807535 (PMC13233499; doi:10.3389/fnhum.2026.1807535)
Supplement: Supplementary file 1 [file Data_Sheet_1.pdf]

Supplementary Material

Letter	Prompt	Letter	Prompt
A	A	P	P
B	B	Q	Q
C	C	R	R
D	D	S	S
E	E	T	T
F	F	U	U
G	G	V	vui
H	H	W	W
I	I	X	X
J	J	Y	Y
K	K	Z	zet
L	L	SPACE	spei
M	emmu	DELETE	delie
N	ennu	COMMA	comma
O	O	PERIOD	peri

Table S1. Text prompts used to generate each letter and symbol stimulus with Amazon Polly. For most letters, the corresponding alphabet character was used as the prompt. However, for letters M, N, V, and Z, alternative prompts (e.g., "emmu", "ennu", "vui", "zet") were used to produce pronunciations that sounded more natural to Japanese listeners. For symbol stimuli, shorter prompts were used (e.g., "spei" for SPACE) to ensure concise audio output, avoiding unnecessarily long utterances.

Runs Group	Run#	Trial 1	Trial 2	Trial 3	Trial 4	Trial 5
Offline	1	T	H	V	F	I
	2	A	X	M	K	U
	3	W	R	G	L	SPACE
	4	N	DELETE	B	D	P
	5	COMMA	S	Q	PERIOD	O
	6	J	E	C	Y	Z
Online	7	E	S	V	D	R
	8	A	B	T	N	L
	9	O	C	I	H	M

Table S2. The target letter for each trial in each run.

Fold #	Training Runs	Test Runs
1	$3, 4, 5, 6$	$1, 2$
2	$1, 2, 5, 6$	$3, 4$
3	$1, 2, 3, 4$	$5, 6$

Table S3. Cross-validation splits used for exploring preprocessing parameters and evaluating classification pipelines (LDA, xdw+LDA, and xdwCov+TS+LDA). Each fold uses four offline runs for training and two for testing.

Fold #	Training Runs	Validation Runs	Test Runs
1	4, 5, 6	3	1, 2
2	1, 2, 6	5	3, 4
3	1, 2, 3	4	5, 6

Table S4. Cross-validation splits used for exploring preprocessing parameters and evaluating the EEGNet4,2 classification pipeline. Each fold uses three offline runs for training, one for validation, and two for testing.

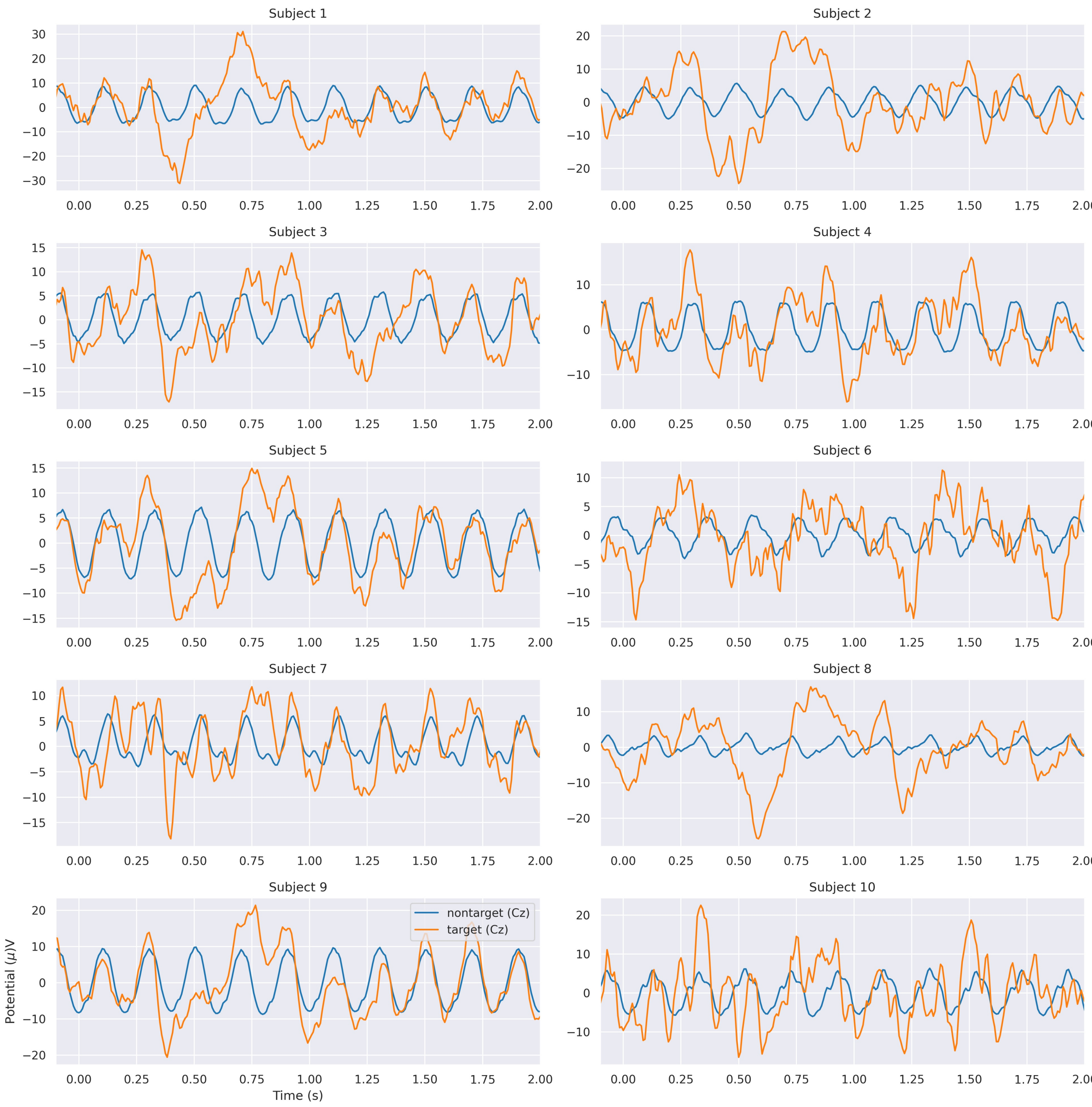

Figure S1. Average ERP responses for each participant at EEG channel Cz. Orange and blue waveforms represent responses to target and nontarget stimuli, respectively. Data from both offline and online runs were used (675 target and 19575 nontarget responses per participant).

1 ANALYSIS OF INTER-PARTICIPANT VARIABILITY IN EEGNET4,2

In Figure 5, different inter-participant variability patterns were observed in EEGNet4,2. In this section, we present additional analyses conducted to investigate the factors underlying this variability.

For each pipeline, analyses were performed using the optimal parameters shown in Table 4. Other preprocessing steps, feature extraction procedures, and model parameters followed the settings described in Section 2.11. Each model was trained using data from the offline runs (runs 1–6). For EEGNet4,2, runs 1–5 were used for training and run 6 was used for validation. Next, the trained models were evaluated on the training data, and the area under the receiver operating characteristic curve (AUC) was computed as the training AUC. For the online runs (runs 7–9), AUC was computed at the trial level using the 450 stimulus-wise classifier outputs within each trial, and then averaged across trials. The generalization gap was defined as the difference between the training AUC and the test AUC (training AUC − test AUC). The mean and inter-participant standard deviation of this difference were then evaluated. The results are summarized in Table S5.

In addition, to investigate the relationship between these metrics and classification performance, Pearson correlation coefficients were computed between the training AUC, test AUC, and their difference (generalization gap), and the letter selection accuracy under the condition of 15 sequences in Section 2.12. The resulting 12 p-values were corrected for multiple comparisons using the Benjamini–Hochberg procedure. The results are summarized in Table S6.

As shown in Table S5, EEGNet4,2 exhibited relatively large inter-participant variance in training AUC, test AUC, and generalization gap, indicating that participant-dependent variability is already present at the binary classification level. Next, we examined the relationship between these metrics and letter selection accuracy (Table S6). Both training AUC and test AUC showed strong positive correlations with classification accuracy, suggesting that performance differences across participants are largely associated with the separability of EEG responses. Regarding the generalization gap, EEGNet4,2 showed a relatively large gap, second only to LDA. However, LDA achieved the highest training AUC (0.91) and test AUC (0.82), suggesting that its larger gap may reflect stronger overall fitting rather than poorer generalization. In contrast, EEGNet4,2 showed lower training AUC compared to LDA, suggesting that the model did not sufficiently fit the training data. This insufficient fitting, combined with potential sensitivity to differences between training and test data, likely contributed to the lower test performance.

Interestingly, EEGNet4,2 achieved very high performance for some participants (e.g., subjects 3 and 7), indicating that the model can perform well when the data characteristics are favorable. This suggests that EEGNet4,2 is more sensitive to participant-specific characteristics than LDA-based methods. One possible explanation for this behavior is the limited amount of training data available for EEGNet4,2. While other models were trained using all six offline runs, EEGNet4,2 used only runs 1–5 for training and reserved run 6 for validation, resulting in fewer samples for parameter optimization. This reduced training data may have limited the model's ability to learn robust representations.

Overall, these results suggest that the variability of EEGNet4,2 is not solely due to overfitting, but is more likely attributable to a combination of insufficient training, limited data, and sensitivity to participant-dependent EEG characteristics. These findings suggest the importance of user-specific optimization, such as tuning hyperparameters (e.g., dropout rate), to fully exploit the potential of deep learning models in this task.

Pipeline	AUC (Train.)	AUC (Test)	Gap
LDA	0.91 ± 0.045	0.82 ± 0.086	0.093 ± 0.059
xdw+LDA	0.82 ± 0.060	0.80 ± 0.096	0.025 ± 0.062
xdwCov+TS+LDA	0.79 ± 0.068	0.78 ± 0.104	0.011 ± 0.058
EEGNet4,2	0.85 ± 0.095	0.80 ± 0.102	0.047 ± 0.067

Table S5. Training and test AUC values, along with the generalization gap (Train − Test), for each classification pipeline. Values are reported as mean ± standard deviation across participants. The generalization gap quantifies the difference between training and test performance, reflecting the extent of overfitting or generalization ability.

Pipeline	AUC (Train.)	AUC (Test)	Gap
LDA	$\mathbf{0.88\,(p = 2.7 \times 10^{-3})}$	$\mathbf{0.94\,(p = 7.8 \times 10^{-4})}$	$\mathbf{-0.69\,(p = 3.5 \times 10^{-2})}$
xdw+LDA	$\mathbf{0.77\,(p = 1.5 \times 10^{-2})}$	$\mathbf{0.88\,(p = 2.7 \times 10^{-3})}$	$-0.63\,(p = 5.8 \times 10^{-2})$
xdwCov+TS+LDA	$\mathbf{0.86\,(p = 3.6 \times 10^{-3})}$	$\mathbf{0.91\,(p = 1.5 \times 10^{-3})}$	$-0.63\,(p = 5.8 \times 10^{-2})$
EEGNet4,2	$\mathbf{0.75\,(p = 1.8 \times 10^{-2})}$	$\mathbf{0.79\,(p = 1.3 \times 10^{-2})}$	$-0.14\,(p = 7.1 \times 10^{-1})$

Table S6. Pearson correlation coefficients between AUC-based metrics (training AUC, test AUC, and generalization gap) and letter selection accuracy. Correlation coefficients are reported with corresponding p-values in parentheses. P-values were corrected for multiple comparisons using the Benjamini–Hochberg procedure. Bold values indicate statistically significant correlations after correction.
